# Supplementary material for: Quercetin treatment reduces the severity of renal dysplasia in a beta-catenin dependent manner
Source: PLoS One. 2020 Jun 17;15(6):e0234375. doi: 10.1371/journal.pone.0234375 (PMC7299361; doi:10.1371/journal.pone.0234375)

Replicate #1

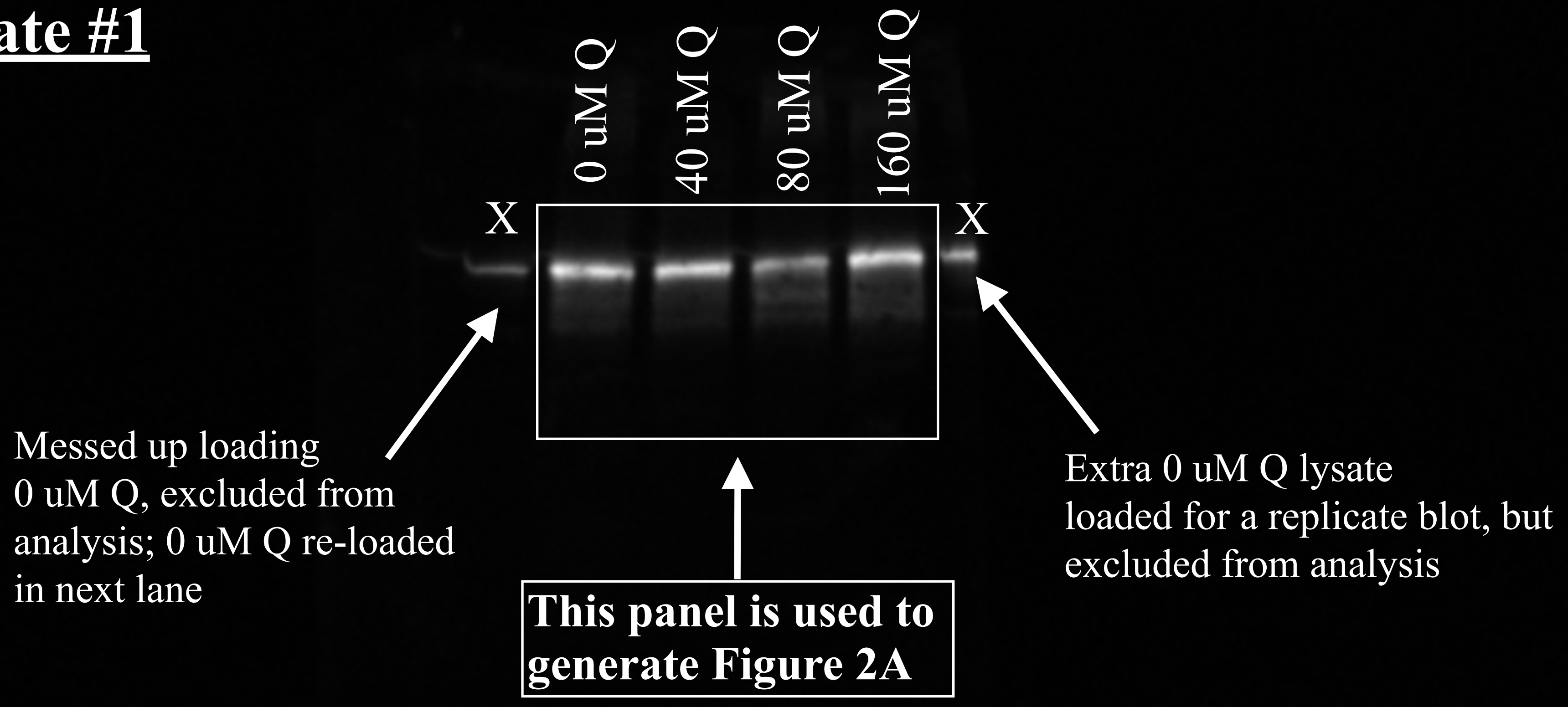

Samples are embryonic mouse kidneys with quercetin treatment (40, 80 and 160 uM Q) and without quercetin treatment as untreated control (0 uM Q). Lysates from at least 10 kidneys per treatment group were each ran on SDS-PAGE and immunoblotted for 3 replicate trials; the bands shown here represent Replicate #1.

Blots were imaged on GeneSys imaging platform, selecting the ‘ECL Chemiluminescence’ option to take an image of the blot. The imaging settings were pre-programmed to have fixed exposure times and contrast levels that will provide the most optimal quality of the bands, and the same settings were applied to all blots across all replicates. Images generated were saved as .TIFF files

These images were analyzed on ImageJ to record densitometry values. For densitometry analysis, all raw blot images were first inverted to make all the bands grey (including any non-specific bands) and the background was set as white. The ‘Mean Grey Value’ of each blot was recorded.

Replicate #2

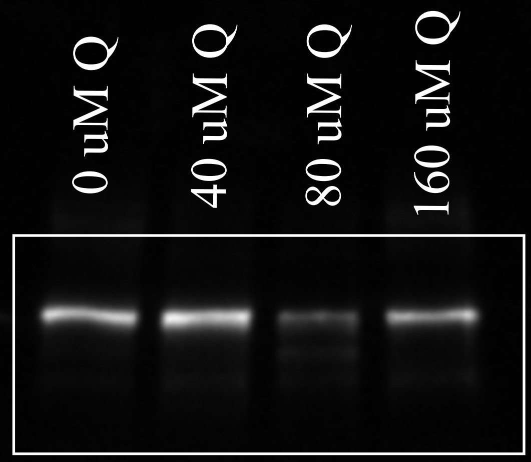

Samples are embryonic mouse kidneys with quercetin treatment (40, 80 and 160 uM Q) and without quercetin treatment as untreated control (0 uM Q). Lysates from at least 10 kidneys per treatment group were each ran on SDS-PAGE and immunoblotted for 3 replicate trials; the bands shown here represent Replicate #2.

Blots were imaged on GeneSys imaging platform, selecting the 'ECL Chemiluminescence' option to take an image of the blot. The imaging settings were pre-programmed to have fixed exposure times and contrast levels that will provide the most optimal quality of the bands, and the same settings were applied to all blots across all replicates. Images generated were saved as .TIFF files

These images were analyzed on ImageJ to record densitometry values. For densitometry analysis, all raw blot images were first inverted to make all the bands grey (including any non-specific bands) and the background was set as white. The 'Mean Grey Value' of each blot was recorded.

Replicate #3

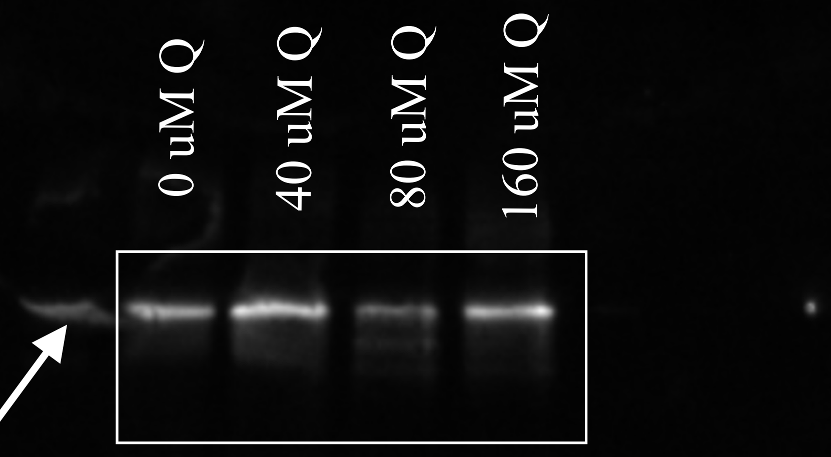

Messed up loading 0 uM Q, excluded from analysis; 0 uM Q re-loaded in next lane

Samples are embryonic mouse kidneys with quercetin treatment (40, 80 and 160 uM Q) and without quercetin treatment as untreated control (0 uM Q). Lysates from at least 10 kidneys per treatment group were each ran on SDS-PAGE and immunoblotted for 3 replicate trials; the bands shown here represent Replicate #3.

Blots were imaged on GeneSys imaging platform, selecting the 'ECL Chemiluminescence' option to take an image of the blot. The imaging settings were pre-programmed to have fixed exposure times and contrast levels that will provide the most optimal quality of the bands, and the same settings were applied to all blots across all replicates. Images generated were saved as .TIFF files

These images were analyzed on ImageJ to record densitometry values. For densitometry analysis, all raw blot images were first inverted to make all the bands grey (including any non-specific bands) and the background was set as white. The 'Mean Grey Value' of each blot was recorded.

Loading control

Samples are embryonic mouse kidneys with quercetin treatment (40, 80 and 160 uM Q) and without quercetin treatment as untreated control (0 uM Q).

Lysates from at least 10 kidneys per treatment group were each ran on SDS-PAGE and stained with Coomassie blue, and imaged on GeneSys imaging platform, using the 'Visible Stain' option to capture the image of the stained gel.

This image was analyzed on ImageJ to record the density of total protein at 45 kDa. Image was first converted to grayscale and the 'Mean Grey Value' of each band at 45 kDa was recorded.

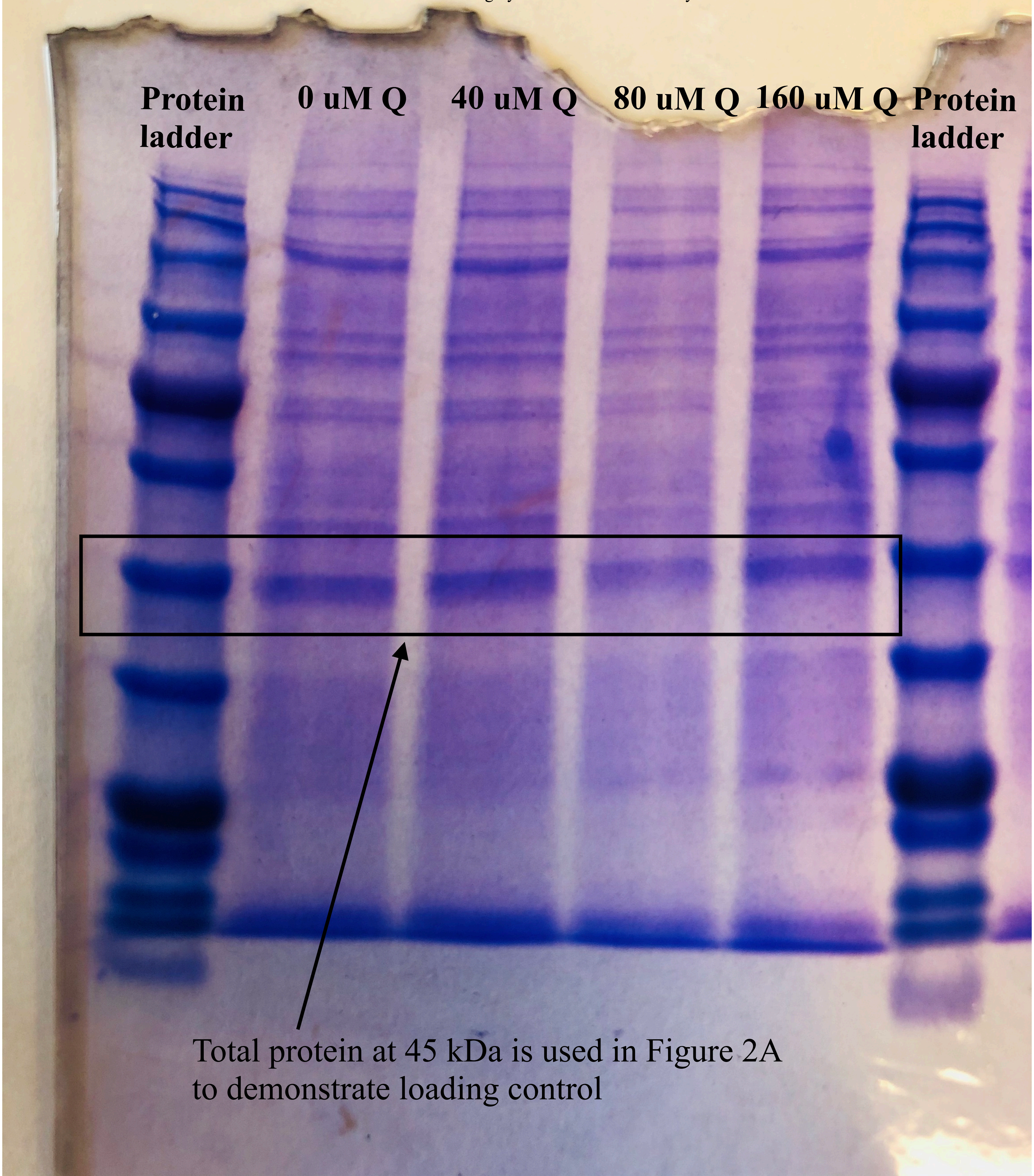

Supplement: S1 Raw Images — PDF file containing all raw, unedited and uncropped TIFF images for gel/blot results used for Fig 2. Three replicates of the Western blot experiment are shown (Replicate #1, #2 and #3). The Coomassie-stained gel used to demonstrate protein loading is also shown. (PDF) [file pone.0234375.s001.pdf]
